# Beneath the surface: expanding the known repertoire of methylotrophic metabolism

**DOI:** 10.1128/aem.02116-25

**Published:** 2026-02-04

**Authors:** Eric L. Bruger, Jannell V. Bazurto

**Affiliations:** 1 Department of Plant and Microbial Biology, University of Minnesota 172728 , Saint Paul, Minnesota, USA; 2 Microbial and Plant Genomics Institute, University of Minnesota 311812, Saint Paul, Minnesota, USA; 3 Biotechnology Institute, University of Minnesota 25793, Saint Paul, Minnesota, USA; Michigan State University, East Lansing, Michigan, USA

**Keywords:** lanthanides, methylotrophs, *Bradyrhizobium*, auxotrophy, XoxF, Calvin-Benson-Bassham cycle

## Abstract

Although the metabolic pathways that allow the utilization of one-carbon compounds as sole sources of carbon and energy (methylotrophy) are well characterized, this understanding has been substantially refined and expanded in recent years. The paradigm-shifting discovery of the lanthanide-dependent methanol dehydrogenase, XoxF, established the biological relevance of rare-earth metals and revealed that methylotrophy required reassessment. We now know that XoxF is broadly distributed among bacteria and may actually constitute an ancestral form by which methylotrophy initially evolved, as well as the predominant form in which it now exists in nature. A new study published in *Applied and Environmental Microbiology* (C. R. Mineo, J. Jiang, and N. C. Martinez-Gomez, 91:e01304-25, 2025, https://doi.org/10.1128/aem.01304-25) extends this knowledge to characterize a heretofore undemonstrated methylotrophic pathway architecture among nitrogen-fixing plant symbionts of the *Sinorhizobium* and *Bradyrhizobium* genera. Their metabolic strategy proceeds via XoxF, complete oxidation to carbon dioxide, and the Calvin-Benson-Bassham cycle to assimilate the oxidized carbon. The authors designate this the “XoxF-CBB pathway,” which appears to be well-conserved across these groups of bacteria. Their streamlined pathway represents a unique connection between autotrophy and methylotrophy that, when paired with XoxF, could constitute an underappreciated, but prevalent, variation on methylotrophy. The study highlights the need to remain open-minded about methylotrophic pathway configurations in bacteria, as well as informing the ways in which we should consider seeking to isolate novel methylotrophs. Finally, the pathway’s presence in nodule-forming bacteria raises new questions about how methylotrophy shapes their physiology in both free-living soil conditions and plant-symbiotic associations.

## COMMENTARY

## LANTHANIDES IN BIOLOGY

In 2005, a methanotroph isolated from a volcanic mudpot in Italy drastically expanded our understanding of single-carbon metabolism, defined as the usage of one-carbon compounds as the sole sources of carbon and energy (i.e., methylotrophy). Its inability to grow independently of the volcanic water eventually revealed a dependence on lanthanide (rare earth) metals as mineral nutrients (1, 2). Within a few years, this dependence was traced back to a specific methanol dehydrogenase (MDH), XoxF, that had long been associated with methylotrophic organisms but whose functional role remained unknown. Soon after, other work characterized the lanthanide-dependence of XoxF-based methanol oxidation in several well-studied aerobic methanol-utilizing facultative methylotrophs, including *Methylobacterium extorquens* and *M. nodulans*, as well as members of *Bradyrhizobium*, which were only recently recognized as methylotrophs in 2011 (3–5). Prior to these discoveries, there were no defined biological roles for lanthanides, so although the *xoxF* gene was noted as a common feature of methylotrophic gene clusters, it had no definitive physiological role. The presence of lanthanides in the volcanic mudpot water and in the subsequent lab-based experiments activated XoxF, revealing new pathways for methanol utilization and methylotrophy in general. This series of observations overturned the longstanding assumption that lanthanides were biologically inert and necessitated a paradigm shift: lanthanides are actually an integral part of organismal metabolism and physiology in various environments. As asserted in the 2015 “Just add lanthanides” perspective by Drs. Cecilia Martinez-Gomez and Elizabeth Skovran, including lanthanides in growth media used for methylotrophic isolations and physiological studies, would vastly expand microbiologists’ understanding of methylotrophy and the role of lanthanides in biological systems. Since then, these authors have done just that, even identifying other lanthanide-dependent alcohol dehydrogenases unrelated to XoxF, expanding the role of lanthanides to multicarbon metabolism (6–8). Recently, Martinez-Gomez’s group has further broadened this framework by comprehensively characterizing the XoxF-lanthanide-dependent methylotrophic pathways conserved in agriculturally relevant *Bradyrhizobium* and *Sinorhizobium* strains. The new report in *Applied and Environmental Microbiology* by Mineo et al. underscores the breadth of this metabolism beyond canonical methylotrophs (9).

## DISTRIBUTION OF LANTHANIDES, METHANOL, AND *xoxF*

Discovering the physiological role of XoxF and the sequence signatures that definitively differentiated it from its calcium-dependent counterpart, MxaF, motivated and enabled studies to identify the natural distribution of XoxF-based methylotrophy (10). Environmental surveys revealed high *xoxF* gene diversity and broad *xoxF* distribution across coastal marine environments, freshwater and marine pelagic ecosystems, lake sediments, shale and weathered granite, underground mine communities, and the phyllosphere (aerial surfaces of plants) in diverse bacteria (11–17). This distribution is consistent with the presence and concentrations of lanthanides and methanol across environments. Lanthanides make up approximately 0.015% of the Earth’s crust by weight, with the most abundant light lanthanide, cerium (Ce, 62 mg/kg), being found at abundances comparable to copper (Cu, 60 mg/kg) (18–20). Lanthanides are predominantly found in the insoluble forms of silicates, carbonates, oxides, phosphates, and oxyhydroxy salts and can accumulate in agricultural soils, the rhizosphere, and in plant tissues (18, 21). These largely insoluble forms, presumed to be biologically inaccessible, were a major reason that lanthanides were thought to be biologically inactive, despite demonstrating significant catalytic potential *in vitro* (22). Critically, environmental concentrations often exceed the minimum lanthanide concentrations determined to be required for growth (2.5 nM lanthanum, La^3+^). Further, lanthanide solubilization and acquisition systems (e.g., lanthanophores) have been characterized, suggesting that lanthanide availability is unlikely to limit XoxF function in most environments (23). Meanwhile, environmental methanol, the second most abundant atmospheric volatile organic compound, is predominantly generated by terrestrial plants and phytoplankton as a byproduct of cell wall (pectin) metabolism in well-characterized diurnal patterns (24, 25). Methanol is a highly soluble gas emitted from plant leaves via their stomata and also excreted via root exudates. Thus, plant-derived methanol is abundant in the phyllosphere and rhizosphere. Interestingly, lanthanides have also been noted to hyperaccumulate in plant roots (21), suggesting colocalization of both lanthanides and methanol is possible in the primary (rhizosphere) and secondary (root nodule symbiosis) habitats of *Bradyrhizobium* and *Sinorhizobium*, potentially enabling the capacity and selection for XoxF-based methylotrophy.

The presence of the *xoxF* gene in methylotrophic genomes was consistently found to be associated with genomic neighborhoods enriched in genes required for methanol and formaldehyde oxidation, implicating XoxF in these processes (26, 27). Given that many organisms queried also had a copy of the *mxaF* gene and that *xoxF* disruptions rarely resulted in detectable phenotypes, its broad significance remained elusive. For example, in well-described model systems for facultative methylotrophy, such as *M. extorquens* AM1 and *Paracoccus denitrificans* (28, 29), both *xoxF* and *mxaF* genes are present, suggesting functional redundancy or differential gene expression under distinct environmental conditions. And although a role for the *xoxF* gene had not been determined in the well-characterized *M. extorquens* AM1 at the time, *in planta* studies yielded the first demonstrable phenotype of the *xoxF* mutant, which was less competitive than the wild-type during colonization, indicating *xoxF* conferred a selective advantage in that particular environment, but not standard laboratory conditions (29, 30). The missing ingredient in laboratory growth conditions were, of course, the presence of lanthanides in the growth medium to activate relevant gene expression (8). The prevalence of genomes containing multiple *xoxF* paralogs and typically a single copy of *mxaF*, along with sequence homologies, suggests that *xoxF* genes are ancestral and that *mxaF* evolved more recently (7, 26, 31, 32). Consistent with this, a number of methane- and methanol-utilizing organisms, such as the novel acidophilic methanotroph isolated from the volcanic mudpot, *Methylacidiphilum fumariolicum* SolV, and the purple non-sulfur photoheterotrophic bacterium *Rhodobacter sphaeroides* are solely reliant on XoxF and do not also possess MxaF, indicating that for many organisms, XoxF is their only available means of methanol oxidation (2, 27). Relatedly, a recent study by Glass et al. identified the most transcriptionally active open-ocean methylotrophs, which rely exclusively on XoxF, prompting the suggested renaming of the alphaproteobacterial order TMED127 to Methylaequorales, to pay homage to their high activity in surface ocean waters (33). Thus, our understanding of the ecological distribution of methylotrophy has evolved over time, and current understanding suggests that lanthanide-driven methylotrophy is more dominant in many environments (11, 17, 31, 34). This revised view of methylotrophic ecology invites renewed investigation into lanthanide-dependent methylotrophy in well-studied, agriculturally and biotechnologically relevant microbes, an opportunity directly addressed by the study discussed here.

## METHYLOTROPHY IN *BRADYRHIZOBIUM* AND *SINORHIZOBIUM*

Members of the genera *Bradyrhizobium* and *Sinorhizobium* are established plant growth-promoting bacteria that provide fixed nitrogen to their plant hosts via root nodulation. Though previously noted to be capable of growth on methanol, the pathways enabling that growth were unknown, and determining them is exactly what Mineo et al. set out to do. Genomic studies have previously identified methylotrophic genes, including *xoxF*, in *Bradyrhizobium* and *Sinorhizobium* strains (12, 15, 31, 35, 36). An early functional investigation in *B. diazoefficiens* USDA 110 (formerly *B. japonicum*) showed weak *xoxF*-dependent methanol oxidation in this strain that could not support growth (37). However, it was not until the discovery of the lanthanide dependence of XoxF MDH enzymes that robust growth on methanol could be observed in *Bradyrhizobium* (38). With the addition of lanthanides, multiple *Bradyrhizobium* strains were found to utilize methanol as a sole carbon source, demonstrating a previously unrecognized capacity for methylotrophic metabolism in this genera. Despite this exciting new finding, the complete set of genes and methylotrophic pathways required for methanol utilization in these organisms remained unknown. Mineo et al. identified the “XoxF-CBB” pathway combination underlying methanol utilization in *B. diazoefficiens* USDA 110, *Bradyrhizobium* sp. USDA 3456, and *S. meliloti* 2011, elucidating the molecular basis of their methylotrophic metabolism. Their work further reverses the consideration of many bacteria designated as non-methylotrophs by traditionally used genetic signatures.

The authors first demonstrated that, like the *Bradyrhizobium* species, *S. meliloti* 2011 can also utilize methanol as a sole carbon and energy source when provided with lanthanides, demonstrating lanthanide-enabled facultative methylotrophy. Further, they use multiple lines of evidence to demonstrate that all three of these facultative methylotrophs use the XoxF-CBB pathway. Specifically, they found that XoxF oxidizes methanol to formaldehyde, a glutathione (GSH)-dependent pathway oxidizes formaldehyde to formate, cellular formate dehydrogenases oxidize formate to CO_2_. Finally, the Calvin-Benson-Bassham (CBB) pathway assimilates methanol-derived CO_2_, demonstrating an independence from atmospheric CO_2_ for biomass generation. Critical for the function of the CBB cycle is the enzyme ribulose-1,5-bisphosphate carboxylase/oxygenase (RuBisCO). Interestingly, RuBisCO is required for efficient symbiosis, specifically influencing colonization and competition for nodulation, but the underlying reason remains unknown (39). The discovery of a RuBisCO-dependent methanol utilization pathway among nodulating bacteria raises the intriguing possibility that this activity contributes to its essential role in symbiosis and/or provides a selective advantage in the presence of lanthanides.

## MODULARITY OF METHYLOTROPHY

Modularity in methylotrophy arises from partitioning the larger metabolic framework into discrete functional units, each achievable through multiple, interchangeable pathways that together constitute this metabolic network. The framework is most commonly broken down into the following functional modules: (i) the “primary oxidation module” where the reduced single-carbon growth substrate (such as methanol, methylated amines, and methylated sulfur compounds) is oxidized to formaldehyde, (ii) the “formaldehyde dissimilation module” where formaldehyde is oxidized, and thus, detoxified, (iii) the “assimilatory module” where the one-carbon (C1) units are incorporated into multicarbon compounds to build biomass (26). For methylotrophy to be possible, each of the modules must be present. As a result of the numerous possible pathway permutations and their overlap with central metabolic and stress response pathways, it can be cumbersome to predict methylotrophic function in organisms solely via genomic analyses. For instance, a related but distinct metabolism that can use common oxidation pathways is methylovory, where the oxidation of C1 compounds is uncoupled from carbon assimilation and used solely for energy (36). Similarly, because formaldehyde is a highly reactive and universally encountered molecule, most organisms have stress response pathways that convert it into less reactive forms for the purpose of detoxification rather than bona fide methylotrophy. Thus, the GSH-dependent formaldehyde oxidation pathway can serve as either for detoxification, as in *Escherichia coli*, or methylotrophic metabolic pathway, as in *P. denitrificans*. There are also several lines of evidence supporting the horizontal transfer of methylotrophic modules, including localization of the methylotrophic genes of *Burkholderia* and *Sinorhizobium* onto symbiotic megaplasmids (9, 40), which thereby allows for extensive variation and adaptation of subparts of the larger network.

Despite many potential combinations of characterized modules, certain permutations appear to be more commonly observed, suggesting that those combinations are either the historical vestige of coevolution, are more selectively favorable, or perhaps are negatively constrained, such as by epistasis. Notably, metabolic modularity is thought to insulate against the effects of negative genetic interactions (41). The principles governing the co-occurrence and compatibility of specific methylotrophic pathways remain an open and compelling question. Points of redundant function are common among some methylotrophic components (e.g., multiple formate dehydrogenases to oxidize formate to CO_2_), and rare among others (e.g., usage of FolD vs MtdA enzymes for formaldehyde oxidation). The nature of pathway configurations favored in a given environment may be dependent upon the specific availability of certain substrates, cofactors, and physiochemical conditions such as temperature and pH. Thus, environmental conditions may favor temporary adaptations, such as switching to a lanthanide-based MDH, or alternatively shape the composition of methylotrophic (sub)populations. In *B. diazoefficiens* USDA 110, methanol utilization required sealed tubes, possibly due to the O_2_ sensitivity of RuBisCO or increased CO_2_ retention; this further illustrates the importance of specific growth conditions in allowing methylotrophic growth and suggesting that, in addition to lanthanides, some microbes may require low-oxygen or anaerobic conditions to effectively utilize methanol (9, 42).

The CBB cycle has rarely been seen as the predominant C1 assimilatory module among methylotrophs prior to the Mineo et al. study, in part due to its relative inefficiency in carbon sequestration (43). Carbon assimilation in methylotrophic bacteria most commonly proceeds through the ribulose monophosphate (RuMP) pathway or the serine cycle where formaldehyde or formate and CO_2_ are assimilated, respectively. In contrast, the CBB cycle, which assimilates CO_2_ following complete oxidation of the C1 growth substrate, appears to be relatively uncommon among methylotrophs as the sole assimilatory pathway. Instead, when present, the CBB cycle is typically found alongside (and was assumed to be auxiliary to) other assimilatory pathways. It has most commonly been observed in methylotrophs among alphaproteobacterial autotrophs (e.g., *P. denitrificans*) and different subsets of methanotrophs (e.g., Verrucomicrobiota and NC10 phyla). Another unique feature of the CBB cycle is that it is shared by non-methylotrophs (autotrophs) and plants, unlike the RuMP or serine cycles.

## THE XoxF-CBB PATHWAY REVEALS AN ANCESTRAL STREAMLINED CONFIGURATION OF METHYLOTROPHIC METABOLISM

The module configuration identified by Mineo et al. is one that has been suggested among methanotrophs (26) but was not yet explicitly reported to exist among extant methylotrophs generally. The authors provide the first direct demonstration of the XoxF-CBB pathway in Rhizobiales using integrated physiological, transcriptomic, and genetic approaches. Combinations of GSH, FDH, and CBB module components had previously been observed but were suggested to be fed by an MxaFI-like MDH (e.g., in the autotrophic *P. denitrificans*) (28). Interestingly, *R. sphaeroides* appears to use a XoxF MDH-fed pathway analogous to that of the Rhizobiales but requires anaerobic light-exposed conditions (42). Genome-based analyses suggested that the methanotroph *Methylacidiphilum infernorum* might couple the XoxF and CBB modules. However, it uses FolD for the oxidation of formaldehyde to formate, rather than the GSH-mediated pathway adopted by *Bradyrhizobium*/*Sinorhizobium* (44, 45). Interestingly, the XoxF-GSH-FDH-CBB module combination may actually be a prevalent natural methylotrophic architecture, especially among soil and rhizosphere-associated microbes, such as nitrogen-fixing Rhizobiales (9). Overall, the XoxF-CBB configuration represents a more streamlined architecture than most described methylotrophic pathways.

Given that both XoxF and CBB are now considered to be among the most ancestral methylotrophic machinery available, the XoxF-CBB pathway may be representative of the simplest and most ancient methylotrophic schema available in nature. XoxF appears to be the more widely distributed MDH across bacterial groups, and this study supports the idea that it can effectively act as the sole oxidation enzyme present to enable functional methylotrophy. Counter to historical assumptions, rather than being an auxiliary player in methylotrophic metabolism, XoxF is now understood to be among the most persistent and ancient methylotrophic modules (though suspected to emerge in Bacteria after diverging from Archaea, unlike the tetrahydrofolate and methanopterin-mediated formaldehyde oxidation modules) (31). Similar to the XoxF oxidation module, the CBB cycle appears to be the sole assimilatory module present among the *Bradyrhizobia* and *Sinorhizobia* strains examined by Mineo et al. It has been postulated that, considering the ancestral nature of RuBisCO and the widespread nature of the CBB cycle, this pathway may have constituted the assimilatory module first adopted by ancestral methylotrophs (26).

## CONCLUSIONS

In the present study, Mineo et al. investigated the impact of plant-derived methanol and lanthanide metals on the growth of nitrogen-fixing Rhizobiales and defined their streamlined methylotrophic pathway. The authors build on existing studies that demonstrated XoxF-dependent methanol oxidation and growth in *Bradyrhizobium* strains, which was suggested to proceed through the CBB cycle, a seemingly uncommon assimilation pathway in methylotrophs that has traditionally been regarded as supplementary (15, 31). Here, the XoxF-CBB pathway architecture identified had a singular oxidation pathway and a single assimilation pathway, bringing to prominence their potential primacy in driving methylotrophy, rather than exclusively serving auxiliary roles. Despite being outlined by the genomic analyses, direct demonstration of the pathway’s function required thorough investigation through a variety of experimental approaches.

The bacteria examined are noted for their symbioses and capacity to enhance growth performance of the leguminous plants they colonize. Although well-characterized in their capacity to fix nitrogen and form nodules, there are still significant gaps in our understanding of the interactions between these beneficial nitrogen-fixers and their host plants as well as a keen interest in understanding and fortifying these interactions. Thus, the activation of methylotrophic pathways via lanthanides brings forth interesting questions about the ecological role of lanthanide-based methanol utilization. Specifically, might lanthanide-activated methylotrophy bolster, or even be required for, plant-bacterial interactions, or perhaps facilitate the survival and/or persistence of free-living bacteria when in soils? Though not widely adopted in the United States, lanthanide fertilizers, noted for their ability to enhance crop growth by unknown mechanisms, have been in use for decades in various countries. A 2025 study found that lanthanides can directly benefit plants by strengthening chlorophylls and protecting plant pigments during UV stress (46). However, lanthanide-based methylotrophy in plant growth-promoting taxa such as the Rhizobiales raises the question of whether lanthanides exert multi-faceted effects, in part by stimulating plant-associated methylotrophs, possibly promoting plant-microbe interactions.

The present study, focused on a breadth of organisms previously considered to be non-methylotrophs, serves as a reminder that we may be consistently underestimating the true prevalence of methylotrophic metabolism in nature, and thereby its ecological roles, such as in biotic interactions and in microbially-mediated global carbon cycling. The complexity of possible methylotrophic pathway configurations and overlap of components with ubiquitous pathways, such as the TCA cycle, complicates efforts to confidently identify methylotrophs based solely on their genomes. Additionally, the current study found that growth conditions required for successfully and broadly identifying bona fide methylotrophs are not trivial. In addition to lanthanides, the XoxF-CBB pathway identified herein required sealed tube conditions to function in one *Bradyrhizobium* species tested. These conditions also improved methanol-based growth for the other two organisms in their study, as well as for *M. extorquens*, which uses a different pathway configuration altogether. Thus, similar to the recognition that adding lanthanides to growth media “unlocked” XoxF-based methylotrophy and expanded our overall view of methylotrophy, environmental studies aiming to isolate representative methylotrophs in a given environment may require a variety of screening conditions to be both successful and comprehensive. Collectively, these observations highlight the expanding roles of methylotrophy across diverse ecological contexts and underscore the need for open-minded exploration of methylotrophic functions, including the likely existence of enzyme and pathway variants that remain uncharacterized to this day.
